# Psychogenic itch

**DOI:** 10.1038/s41398-018-0097-7

**Published:** 2018-03-01

**Authors:** Laurent Misery, Sabine Dutray, Myriam Chastaing, Martine Schollhammer, Sylvie G. Consoli, Silla M. Consoli

**Affiliations:** 1 French Psychodermatology Group, French Society of Dermatology, Paris, France; 2 Department of Dermatology, University Hospital of Brest, Brest, France; 3 Laboratory of Neurosciences of Brest, University of Western Brittany, Brest, France; 4 Department of Psychiatry and Medical Psychology, Unit of Liaison Psychiatry, Brest, France; 5 Department of Consultation Liaison Psychiatry, Paris-Descartes University, Sorbonne Paris Cité, Paris, France

## Abstract

Psychogenic itch can be defined as “an itch disorder where itch is at the center of the symptomatology and where psychological factors play an evident role in the triggering, intensity, aggravation, or persistence of the pruritus.” The disorder is poorly known by both psychiatrists and dermatologists and this review summarizes data on psychogenic itch. Because differential diagnosis is difficult, the frequency is poorly known. The burden is huge for people suffering from this disorder but a management associating psychological and pharmacological approach could be very helpful. Classification, psychopathology, and physiopathology are still debating. New data from brain imaging could be very helpful. Psychological factors are known to modulate itch in all patients, but there is a specific diagnosis of psychogenic itch that must be proposed cautiously. Neurophysiological and psychological theories are not mutually exclusive and can be used to better understand this disorder. Itch can be mentally induced. Opioids and other neurotransmitters, such as acetylcholine and dopamine, are probably involved in this phenomenon.

## Introduction

Psychogenic itch is a diagnosis that is (too) frequently proposed by physicians, but patients diagnosed with psychogenic itch are (too) rarely referred to psychiatrists. Only 62 references containing the key word “psychogenic itch” could be obtained from PubMed in July 2017. Psychogenic itch is also known as psychogenic pruritus, somatoform pruritus^1^, functional itch disorder^2^, or functional pruritus, but “psychogenic itch” is the most commonly used denomination.

In this review, data on itch will be first provided. Then epidemiology and definition, as well as differential diagnosis and inclusion in classifications will be discussed. Physiopathological and psychopathological points of view will be debated. Finally, the consequences of psychogenic itch and its management will be exposed.

## What is itch?

Itch is only related to the skin and some mucosa^3^. Its definition has remained unchanged for more than 350 years^3,4^: an unpleasant sensation that leads to the need to scratch. Itch is not a slight pain. Although itch and pain are sometimes associated with one another, and both may be associated with other symptoms (burning, stinging, and tingling), itch and pain are different symptoms. Selective pruriceptors have been located in the skin. Specific pathways of pruritus from the skin (around the dermo-epidermal junction) to the brain have also been identified. Many mediators are involved in pruritus, but at minimum, there is a histaminergic and a non-histaminergic pathway (PAR-2-dependent). Gate control or peripheral and central sensitization mechanisms have been highlighted in pruritus, similar to those associated with pain. In the brain, the perception of itch is not restricted to the sensory areas but requires the cooperation of sensory, motor, and affective areas, and the precuneus plays a specific role. Numerous pathophysiological advances have occurred recently and are promising for therapeutic advances, which will be very useful for the symptomatic treatment of pruritus (present treatments are poorly efficient).

The International Forum for the Study of Itch (IFSI), which is the international society dedicated to itch, considers the terms “itch” and “pruritus” synonymous and defines six etiological categories of itches: dermatological, systemic, neurological, psychogenic, mixed, and “other^5^.” Skin diseases are by far the leading cause of itch, but other causes are also possible; such causes may have no origin in the skin itself or in skin lesions associated with itch. Neuropathic itch refers to pruritus caused by neuronal or glial damage^6^, whereas psychogenic itch is related to psychological disorders^7^. Hence, psychogenic itch is a category of chronic itch.

Itch is a common symptom: one-third of the population experiences itch each week, and 10% of the population requires itch treatment^3^. Itch strongly alters the quality of life and is frequently followed by psychiatric comorbidity, including suicidal ideations^3^.

## The burden of itch

Like pain, nausea or asphyxiation, itch can cause major suffering. A large epidemiological study (4995 participants) of the burden of common skin conditions in dermatological patients across Europe^8^ showed that compared with participants who did not report itch, those who reported itch showed more limitations on the European Quality of life-5 Dimensions questionnaire (EQ5D, a generic instrument) and a larger impact on the dermatology life quality index (DLQI), 60% vs 25%. The negative impact on health-related quality of life (HRQoL) resulting from chronic itch increases with the intensity of the pruritus^9^. This relationship is not necessarily a linear one but is dependent on various factors, such as body site, coping abilities, and personality.

The negative impact of itch on quality of life has consequences on mental health^9^. Many studies have found a positive association between itch and depression scores. Patients who scored high on a depression scale, also scored higher for itch intensity than patients, who scored low in depression scales^4^.

Anxiety and depression are consequences of itch as well as aggravating factors for itch condition and scratching. Psychogenic factors frequently enhance somatic sensations, such as pruritus or pain^10^ and neither psychogenic nor organic pruritus could exist in a pure form^11^. Hence, the broad majority of patients with pruritus suffer from a somatic disease and their symptoms can be modulated by psychological factors, such as depression or anxiety. Nonetheless, some patients have a specific psychogenic pruritus.

## The frequency of psychogenic itch

Psychiatrists commonly consider psychogenic itch a very rare condition because these patients usually prefer to consult dermatologists and avoid psychiatrists, as they initially associate the sensation with the skin rather than with a psychological suffering. In contrast, the majority of dermatologists are convinced of the reality of psychogenic pruritus and frequently proposes this diagnosis for patients. The only epidemiological study of psychogenic itch reported that 6.5% of outpatients from a university department of dermatology suffered from “somatoform pruritus^12^,” but that department is known to specialize in psychosomatic dermatology.

In our opinion, psychogenic itch is unfortunately too often mislabeled as idiopathic pruritus because the patient is anxious or the doctor has no other diagnosis to propose. Think of Nanni Morretti film “Caro Diaro” (Dear Diary): In this movie, the patient was misdiagnosed with psychogenic itch for many years and then finally diagnosed with Hodgkin’s disease. Hence, an excessively rapid (mis)diagnosis may have severe psychological and medical consequences for the patient.

## Definition

At the individual level, patients need an adequate diagnosis. At the collective level, a better understanding of psychogenic itch is only possible through clinical and physiopathological studies using diagnostic criteria. Consequently, it is crucial to use a precise definition for correct diagnosis and research.

The French Psycho-Dermatology Group (FPDG) is an expert group from the French Society of Dermatology that includes dermatologists, psychologists, and psychiatrists. This group has proposed a definition of psychogenic pruritus as “an itch disorder where itch is at the center of the symptomatology and where psychological factors play an evident role in the triggering, intensity, aggravation, or persistence of the pruritus” and has suggested calling it “functional itch disorder” (FID)^2^.

In place of the term “psychogenic pruritus,” the FPDG^2^ discussed other possibilities, such as “non-organic pruritus,” “psychosomatic pruritus,” “somatoform pruritus,” “itch disorder associated with psychological factors,” and “functional itch disorder.” The FPDG preferred “functional disorders” to “somatoform disorders” because the consensual opinion was that there is neither a somatic nor a psychiatric diagnosis underlying FID, although an internal psychological conflict is possible. In contrast, the term “functional disorders” suggests a medical definition for a disorder with no apparent somatic cause but for which an associated mental disorder or disease is possible. An associated psychological conflict preceding the onset of the symptoms or a psychiatric disorder may not necessarily be found when FID is diagnosed but may be revealed later.

The IFSI uses the term “somatoform pruritus^5^.” European guidelines for itch favor the phrase “somatoform itch,” which is convenient for easier international use and avoids the word “psychogenic^12^.” In any case, all of these words pertain to pruritus with psychological factor(s) as the main cause. Some major international classifications do not give precise definitions (see below) but situate psychogenic itch within groups of diseases, which could also be helpful. Because “psychogenic itch” remains the more commonly used term, we will use it in this review.

Psychogenic itch is not an idiopathic pruritus (pruritus of unknown origin), and it is not an elimination diagnosis. To assess the diagnosis, it is necessary to determine both negative (no somatic cause) and positive criteria (clinical characteristics, association with psychological disorders, or stressful life events). The FPDG proposed 10 diagnostic criteria (3 compulsory and 7 optional) in its definition^2^ (Table 1). Hence, the diagnosis of FID is possible in the presence of the three compulsory criteria and at least three out of seven of the optional ones. These criteria were validated by an international study that found that the effects of psychotropic drugs and psychotherapies were less able to discriminate FID from other itch diagnoses^13^.

**Table 1:** Diagnostic criteria for functional itch disorder (or psychogenic pruritus) from the French Psychodermatology Group (previously published in *Acta Derm. Venereol*. 2007;87:341–4)

Three compulsory criteria:
Localized or generalized pruritus sine material (without primary skin lesion)
Chronic pruritus (>6 weeks)
No somatic cause
Three of seven optional criteria:
A chronological relationship between the occurrence of pruritus and one or several life events that could have psychological repercussions
Variations in intensity associated with stress
Nycthemeral variations
Predominance during rest or inaction
Associated psychological disorder
Pruritus that could be improved by psychotropic drugs
Pruritus that could be improved by psychotherapies

Psychogenic itch may accompany other psychiatric conditions like depression, anxiety, obsessive-compulsive disorders, psychoses, and substance use^9,14,15^.

## Differential diagnosis

In some patients, authentic somatic pruritus can be associated with psychogenic pruritus or can be aggravated by psychological factors that cannot be identified upon initial visit. All causes of pruritus^4^ are differential diagnoses; this is especially true of non-dermatological itches because in many cases, there are no visible lesions other than those caused by scratching. The aggravation of unpleasant sensations in the evening and the night is common to all etiologies of itch and could be confused with psychogenic itch. Atypical manifestations of itch, especially associations with other symptoms, such as pain, allodynia, paresthesias, hyperesthesia, or hypoesthesia, and/or electric shock sensations, are too often mistaken for manifestations of psychogenic itch, whereas they are actually indicative of neuropathic itch^6^. This is especially true of recently described and poorly known diseases, such as small-fiber neuropathies^16^. In any case, an examination to identify a somatic cause of pruritus is compulsory^4^. Other differential diagnoses of psychogenic pruritus are psychogenic urticaria and psychogenic dermographism, but visible urticarial lesions are observed in patients with those diagnoses.

The main differential diagnosis is discussed in patients who scratch themselves in the absence of itch, those for whom itch is not the main cause of the scratching, and those whose scratching is disproportionate to the intensity of the itch. In these cases, the central symptom is not itch but scratching, and we are in the field of self-inflicted skin lesions (SISL)^17^. It is of particular interest to separate psychogenic excoriations^18^, dermatitis artefacta, and all other SISLs^17^ from psychogenic pruritus. SISLs are related to impulsive, compulsive, or other psychopathological mechanisms. In contrast, psychogenic pruritus is related to an illusion of pruritus, but this pruritus is felt by the patient and is the main complaint. Skin excoriations can be performed by other people, especially the parents in cases of children; we must keep in mind that skin excoriations are sometimes not self-inflicted and can be abusive, such as in Silvermann or Munchausen by proxy syndromes.

Delusional infestations^18^ (Ekbom syndrome, Morgellons syndrome and others) can also be discussed with psychogenic itch. These patients sometimes report itch or crawling sensations, but their main complaint is centered on infestation, and itch is not the main symptom.

Finally, psychogenic itch can be considered to belong to a family of disorders that we suggest calling “functional muco-cutaneous disorders” or “somatoform muco-cutaneous disorders.” This family includes disorders such as cutaneous psychogenic pain or paresthesia and some cases of vulvodynia, scrotodynia, penodynia, stomatodynia, glossodynia, trichodynia, and reactive/sensitive/hyperreactive/irritable skin. These disorders are similar to other disorders that are not in the muco-cutaneous domain, such as psychogenic pain, psychogenic cough, and irritable bowel syndrome. Fibromyalgia and multiple chemical sensitivities could be added to this broad family of medically unexplained syndromes (MUPS)^19,20^. Psychogenic itch is sometimes associated with other somatoform disorders, but the specificity of psychogenic itch among all these disorders is the presence of a localized or generalized itch in comparison with other symptoms, according to the FPDG criteria^2^.

## Classification

Regarding international classifications of psychiatric diseases, psychogenic pruritus is not cited in the 10th revision of the International Classification of the Diseases (ICD-10)^21^; however, pruritus is reported in the diagnosis “other somatoform disorders” (F45.8) along with dysmenorrhea, dysphagia, psychogenic stiff neck, and bruxism. These disorders are classified among somatoform disorders, which are included in the broader category “neurotic disorders, stress-linked disorders and somatoform disorders.” The 11th revision (ICD-11) is due by 2018.

The term “psychogenic pruritus” was not used in the 4th edition of the Diagnostic and Statistical Manual of Mental Disorders (DSM-IV)^22^, but it could be recognized among the following four diagnoses listed in the DSM-IV:

- Conversion disorder (300.11): Unexplained symptoms or deficits affecting voluntary motor or sensory function that suggest a neurological or other general medical condition. Psychological factors are judged to be associated with the symptoms or deficits.

- Undifferentiated somatoform disorders (300.81): One or several somatic complaints lasting six months or more with no medical or mental disease available to explain the presence or intensity of these symptoms. This symptom is not intentionally self-induced or simulated.

- Unspecified somatoform disorder (300.82): All disorders with somatoform symptoms that do not fit the criteria of any specific somatoform disorder.

- Pain disorder associated with psychological factors (307.80): Psychological factors play a critical role in the triggering, intensity, aggravation, or persistence of the pain.

The 5th edition of the Diagnostic and Statistical Manual of Mental Disorders (DSM-5)^23^ replaces somatoform disorders with somatic symptoms and related disorders and makes significant changes to the criteria to eliminate overlap across the somatoform disorders, clarify their boundaries and better reflect the complex interface between mental and physical health. The DSM-5 defines a new group of “somatic symptom disorders” (SSD), which is characterized by “somatic symptoms that are either very distressing or result in significant disruption of functioning, as well as excessive and disproportionate thoughts, feelings and behaviors regarding those symptoms. To be diagnosed with SSD, the individual must be persistently symptomatic (typically at least for 6 months).”

The DSM-IV disorders of somatization disorder, hypochondriasis, pain disorder, and undifferentiated somatoform disorder have been removed, and many, but not all, individuals previously diagnosed with one of these disorders could now be diagnosed with SSD. The DSM-IV diagnosis of somatization disorder required a specific number of complaints from among four symptom groups. The SSD criteria no longer have such a requirement; however, somatic symptoms must be significantly distressing or disruptive to daily life and must be accompanied by excessive thoughts, feelings, or behaviors.

Another key change in the DSM-5 criteria is that while medically unexplained symptoms were a key feature for many of the disorders in the DSM-IV, an SSD diagnosis does not require that the somatic symptoms are medically unexplained. In other words, symptoms may or may not be associated with another medical condition. The DSM-5 narrative text description that accompanies the criteria for SSD cautions that it is not appropriate to diagnose individuals with a mental disorder solely because a medical cause cannot be demonstrated. Furthermore, regardless of whether the somatic symptoms can be medically explained, the individual would still have to meet the remaining criteria to receive a diagnosis of SSD.

Although debatable^24^, this new classification and the new diagnosis of SSD have been validated by clinical studies^25^ and long discussions. Concerning pruritus, SSD includes both psychogenic pruritus and pruritus of a somatic origin with a disproportionate resounding. In our opinion, the concept of SSD is very confusing because it merges both psychogenic itch and severe psychological consequences of any itch. As in the case of psychogenic pain^26^, the concept of SSD overpsychologizes people with chronic pain and may contribute to misdiagnosis and unnecessary stigma. The diagnosis of SSD requires taking a full history, including a review of systems^27^. In contrast, one study suggests that for a population of patients with medically unexplained physical symptoms (MUPS), the DSM-5 SSD criteria are more restrictive than the DSM-IV criteria for somatoform disorders^28^.

Psycho-dermatological classifications (associated skin and psychological disorders) have included pruritus sine materia among “psychological disorders responsible for skin sensations^29^,” “functional cutaneous and mucous disorders^30^” or “conditions in which strong psychogenic factors are imputed^31^.”

## Physiopathology

As reported above, the brain is a crucial organ for itch: the perception of itch is obviously not possible without the brain, and itch can sometimes originate in the brain in patients with disorders of the central nervous system^6^ and those with psychological disorders. In the brain, sensory, motor, and affective areas are activated at the same time when pruritus occurs and even when we think about pruritus or scratching^32–35^. Hence, a new definition of pruritus could be “a sensation accompanied by the contralateral activation of the anterior cortex and the predominantly ipsilateral activation of the supplementary motor areas and the inferior parietal lobule; scratching may follow^36^,” reflecting the fact that “it is the brain that itches, not the skin^37^.” This very important role of the brain in the pathogenesis of pruritus confirms that a psychological component could be present in every case of pruritus^38^ and that a specific psychogenic pruritus is possible^37^. Itch can be mentally induced^39^. Opioids^40^ and other neurotransmitters, such as acetylcholine^41^, are probably involved in this phenomenon.

People with psychogenic pruritus (or other causes of itch) frequently scratch more and more, inducing nerve hyperplasia in the skin and further pruritus. Transient scratching inhibits the itch sensation, but chronic and increasing scratching is common and is related to peripheral and central sensitizations to itch^37,42–44^. These phenomena are similar to sensitizations to pain. Inflammatory mediators are released by scratching-sensitized pruriceptors (peripheral sensitization), whereas this chronic skin inflammation facilitates spinal and cerebral itch processing, resulting in touch-evoked pruritus (central sensitization). The understanding of central sensitization for itch improves our understanding of psychogenic pruritus.

The “contagious itch” has been experienced by all readers of this paper. Witness our neighbor scratching, discussing or reading about itch, watching movies showing people scratching and viewing pictures of affected skin or insects can induce itch in healthy persons and to a greater degree in chronic itch patients and subjects with neurotic personalities^9^. The underlying course of contagious itch is not yet fully understood. It is hypothesized that there are human mirror neurons that are active when we imitate actions and/or negative affect^45^. As evidenced by brain imaging, there is an important functional coupling of the insula and basal ganglia in initiating the urge to scratch when provided with itch-evoking visual stimuli^46^.

An overactive limbic system, particularly the anterior cingulated cortex, which is essential in modulating emotional and cognitive activities, may be clue to our underlying desire to itch^9^. The co-activation of the prefrontal cortex in conjunction with the limbic system upon application of itch stimuli suggests interplay of this network on motivation and emotion^47^. Moreover, the role of the prefrontal cortex has been highlighted on reward processing in addictive behaviors^9^. The reward circuit, particularly in the midbrain, may be part of the neural basis of the vicious circle itch-scratch-itch and appears to be linked to the development of an urge to scratch to achieve the “pleasure” derived from scratching^46^. The involvement of midbrain strongly suggests that there is a role for the dopaminergic system in the addictive nature such a circle^48^.

While there have been no prospective studies on this subject, some experimental and cross-sectional studies indicate that stress factors can influence itch^42^. Perceived stress also affects the capability of healthy subjects to discriminate among itch stimuli^43^, probably by decreasing gate control. Major and minor life events have been shown to be associated with higher levels of itch in the general population and in patients with skin diseases^43^. Stress induces the release of many mediators, and these mediators are responsible for enhancing itch after stress. Many mediators are implicated in enhancing itch after stress. Among them, β2-adrenoceptors mediate itch hypersensitivity following chronic stress by inducing proinflammatory factors, such as TNF-α, in the skin.

## Psychopathology

Personality characteristics and depression could be identified as predictors of experimentally induced itch. Patients who scored high on depression measures reported higher degrees of induced itch compared with patients who reported not being depressive. Furthermore, more than 50% of the variance in induced scratching movements could be predicted by agreeableness and public self-consciousness: patients who reported not being very agreeable and who scored high on public self-consciousness showed a higher increase in the number of scratching movements compared with agreeable patients who did not care much about what others thought about them^44^.

The concepts of the Skin-ego (*Moi-peau*)^49^, somatoform dissociation^19^, and coping^50^ could be useful for the understanding of psychogenic itch.

The *Moi-peau* designates a fantasized reality that a child uses during its early development to represent itself as “me” based on its experience of the body surface; this representation is completed along the child’s lifespan. The child, enveloped in its mother’s care, fantasizes of a skin shared with its mother: on one side is the mother (the outer layer of the *Moi-peau*), and on the other side, the child (the inner layer of the *Moi-peau*). These two layers must separate gradually if the child is to acquire its own ego-skin^51^. However, the ego remains partly identified with the skin. This theory helps to understand why psychological conflicts may be translated into skin symptoms. Psychogenic itch could be a symptom of the patient feeling his or her own limits.

Dissociation is defined in the DSM-IV as a disruption in the usually integrated functions of consciousness, memory, identity, or perception of the environment. Symptoms of psychological and somatoform dissociation are correlated. Itching appears as a symptom of somatoform dissociation, and even milder degrees of dissociation may play a role in its genesis^19^.

Coping is widely accepted as the “efforts, both action-oriented and intrapsychic, to manage (i.e., master, tolerate, reduce, and minimize) environmental and internal demands, and conflicts among them, which tax or exceed a person’s resources.” Coping may serve one of the following functions: problem solving or emotional regulation^52^. Coping has been shown to be a mediator of the relationship between stress and itch in patients with atopic dermatitis^50^.

More global approaches have been proposed for somatoform disorders^49^. In the cognitive-behavioral model, the hallmark is the assumption of perceived bodily sensations that are interpreted in a catastrophic manner, thereby increasing arousal and the probability of intense bodily sensations that are, again, misinterpreted as harmful. In the cognitive-psychological model, the perception of itch should be compared with illusions related to dysfunctions of the primary attentional system followed by the development of chronicity related to dysfunctions of the secondary attentional system.

The psychobiological model is a synthetic model^49,50^ that suggests that psychogenic itch should be the consequence of two main phenomena: first, an increase in body signals caused by numerous (mostly biological) factors resulting from frequent distress, a lack of physical condition or a chronically stimulated hypothalamic-pituitary-adrenal axis; second, deficient gate control that amplifies bodily signals rather than inhibiting or effectively selecting them as it would in healthy people.

## Consequences of psychogenic itch

### Skin lesions

**Fig. 1:**
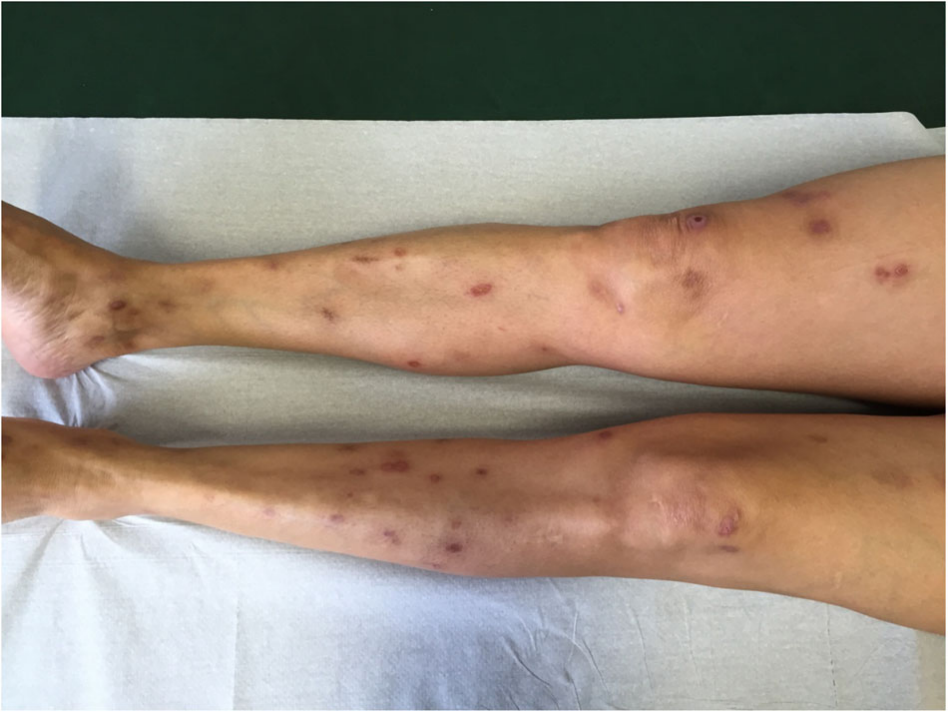
Scratching lesions induced by psychogenic itch

In some patients, there is no scratching; however, usually psychogenic itch is accompanied by scratching, of which patients are more or less aware. The induced lesions (Fig. 1) are commonly found on the body areas that are most accessible to the hands, such as superior and inferior limbs, shoulders, abdomen, scalp, neck, and face. The back is minimally involved, especially the upper parts that are difficult to reach, and a “butterfly area” without no scratching lesions is considered suggestive of psychogenic itch. The excoriations can be superficial or deep, and their size and shape are highly variable. With the chronicity of scratching, the skin becomes thickened and hyperpigmented, and nodules or hypopigmented atrophic scars can occur. Usually, patients use their nails, but some use a variety of objects. Therefore, the lesions may be painful or become infected.

### Burden

The psychogenic origin of itch does not diminish the burden of itch. Itch, including psychogenic itch, causes considerable physical and psychological distress, adversely affecting quality of life and inducing psychiatric comorbidity^53–55^.

Itch is obviously unpleasant and is not associated with pleasure. Like pain, pruritus induces suffering and never pleasure, although scratching can sometimes provide a transitory pleasant feeling. This is why a vicious itch/scratch/itch cycle is common. The hedonic experience is not related to the itch but to scratching, as confirmed by studies^42^ showing that scratching actives hedonic cerebral areas, releasing opioids and inducing itch. Moreover, itch and scratching can be induced purely by visual stimuli or a public lecture on itching^39^. Hence, itch is contagious not only for patients but for those around them!

## Management

### Announcement of diagnosis

The announcement of a diagnosis of psychogenic pruritus to a patient is not easy or anecdotal and should be made cautiously^56^. First, a diagnosis that has been supported by diagnostic criteria (Table 1) to avoid misdiagnosis is essential. There is a need to propose the diagnosis as the more probable hypothesis and to be cautious. Some patients could feel guilty about their itch if they are told that it is simply psychological. To prevent this, it is necessary to talk about this possible diagnosis at the first consultation for a pruritus without dermatological disease and to explain that itch can originate in organs other than the skin, including the brain. After clinical, biological, and radiological exams and conversations to become better acquainted with the patients, this diagnosis will be naturally inferred or confirmed, like any other diagnosis. It is important to explain to the patient that he or she is not responsible for the initiation of the itch and to approach a patient with psychogenic pruritus with the same objectively derived list of differential diagnoses and the same comprehensive treatment plan that would be given any other patient. Patients need to be told and to feel that their suffering is genuinely understood.

### Pharmacological treatment

There has been no clinical trial of treatments for psychogenic itch^57^, and the course of the disease is poorly known. The following psychopharmacologic drugs might be useful and have an acceptable risk of adverse events: hydroxyzine, tricyclic antidepressants (mainly doxepin), and selective serotonin re-uptake inhibitors (fluoxetine, sertraline, paroxetine, citalopram, fluvoxamine, and escitalopram)^58^. Antipsychotics (such as pimozide or risperidone and olanzapine) and antiepileptics (such as topiramate, gabapentin, and pregabaline) can also be used in some cases (7, 52^57^). The choice among these drugs may be discussed according to the general psychiatric context and the eventually associated psychiatric symptoms.

### Psychological approach

Any psychological intervention implies that the symptom is accepted as real and distressing for the patient^49^. An interesting three-level approach has been proposed by Fried^11^ and includes the lesional, emotional, and cognitive levels. In all patients, scratching lesions and prurigo are treated. The emotional level approach can be made through doctor–patient alliance and emotional support followed by personalized psychoanalysis, psychotherapies, hypnosis, or behavioral therapies. Patients’ cognitions need to be improved through an understanding of their disease and the absence of guilt, an appropriate attitude toward washing and alternatives to scratching (therapeutic education).

Contrary to some other functional somatic syndromes, the efficacy of psychological interventions for psychogenic itch has not been evaluated^49^. Interventions aimed at stress reduction and relaxation and training in problem solving should be beneficial^49^. As a starting point, the formulation of a biopsychosocial model may be helpful. Cognitive-behavioral therapy could interrupt the vicious itch-scratching circle^49^.

## Conclusions

Although psychological factors can modulate itch in all patients, the specific diagnosis of psychogenic itch must be proposed cautiously. Currently, research on somatoform disorders is oriented according to the somatosensory amplification theory: aberrant interactions across neural circuits mediating visceral-somatic perception, emotional processing/awareness, and cognitive control are proposed to serve important roles in the neurobiology of somatosensory amplification^53^. Hence, neurophysiological and psychological factors are not mutually exclusive and can be coordinated for the patient’s benefit. The recent more precise definitions of psychogenic itch will be very helpful for more accurate research.
